# Heterogeneity of colorectal cancer risk by tumour characteristics: Large prospective study of UK women

**DOI:** 10.1002/ijc.30527

**Published:** 2017-01-18

**Authors:** Andrea Burón Pust, Rupert Alison, Roger Blanks, Kirstin Pirie, Kezia Gaitskell, Isobel Barnes, Toral Gathani, Gillian Reeves, Valerie Beral, Jane Green, Million Women Study Collaborators

**Affiliations:** ^1^ Nuffield Department of Population HealthCancer Epidemiology Unit, University of OxfordUnited Kingdom; ^2^ Epidemiology and Evaluation DepartmentHospital del MarBarcelonaSpain; ^3^ IMIM (Hospital del Mar Medical Research Institute)BarcelonaSpain; ^4^ REDISSEC, Health Services Research on Chronic Patients NetworkMadridSpain

**Keywords:** colorectal cancer, risk factors, subtype, smoking

## Abstract

Associations between behavioural and other personal factors and colorectal cancer risk have been reported to vary by tumour characteristics, but evidence is inconsistent. In a large UK‐based prospective study we examined associations of 14 postulated risk factors with colorectal cancer risk overall, and across three anatomical sites and four morphological subtypes. Among 1.3 million women, 18,518 incident colorectal cancers were identified during 13.8 (SD 3.4) years follow‐up *via* record linkage to national cancer registry data. Cox regression yielded adjusted relative risks. Statistical significance was assessed using correction for multiple testing. Overall, colorectal cancer risk was significantly associated with height, body mass index (BMI), smoking, alcohol intake, physical activity, parity and menopausal hormone therapy use. For smoking there was substantial heterogeneity across morphological types; relative risks around two or greater were seen in current smokers both for signet ring cell and for neuroendocrine tumours. Obese women were also at higher risk for signet ring cell tumours. For adenocarcinomas, the large majority of colorectal cancers in the cohort, all risk factor associations were weak. There was little or no heterogeneity in risk between tumours of the right colon, left colon and rectum for any of the 14 factors examined. These epidemiological findings complement an emerging picture from molecular studies of possible different developmental pathways for different tumour types.

## Abstract 2

**What's new?**

Evidence suggests that risk factors are differentially linked to colorectal cancer type and tumor site, suggesting that the disease can arise *via* distinct pathways, likely depending on the type of precancerous lesion. To explore risk factor associations by anatomical site and tumor morphology, the authors of this study examined data from the Million Women Study cohort, with more than 18,500 incident colorectal cancer cases. While there was little evidence for risk differences by tumor site, substantial differences were found for tumor morphology, particularly for smoking, which showed relative risks of twofold or more for some rare colorectal tumors.

It has been suggested that risk factors for colorectal cancer may vary by tumour site, and by morphological type, possibly reflecting different pathways by which tumours develop from conventional adenomas or serrated bowel polyps.^1^, ^2^, ^3^, ^4^ Much existing evidence is inconsistent and limited by small numbers of cases in most individual studies. Studies need to be very large to have enough power to detect reliably heterogeneity in risk by colorectal cancer site or by tumour subtype.

We report here on associations observed between certain behavioural and personal characteristics and incident colorectal cancer risk, comparing risks across anatomical site and across morphological subtype, in a prospective study of over 1 million UK women.

## Methods

### Study population, data collection and follow‐up

Between 1996 and 2001, around 1.3 million women aged on average 56 years (SD 5) joined the Million Women Study through National Health Service (NHS) breast screening clinics in England and Scotland, completing a questionnaire about anthropometric, social and demographic factors, and other personal characteristics. The cohort has been resurveyed every 3 to 5 years since then. The study design and methods are described in detail elsewhere,^5^, ^6^ and questionnaires can be viewed online at http://www.millionwomenstudy.org.

All participants gave consent for follow‐up through their medical records, and have been linked to the NHS Central Register; study investigators are routinely notified of deaths, emigration and cancer registrations through the Health and Social Care Information Centre in England, and the Information Services Division in Scotland. Follow‐up data were available for England to December 31, 2013, and for Scotland to December 31, 2008.

To date, only about 1% of the cohort has been lost to follow‐up, mainly by emigration and cessation of registration with the NHS; such women contribute person‐years and events up to the date when follow‐up ceased.

The information provided for cancer includes the date of diagnosis together with the cancer site (coded using the 10th revision of the International Classification of Diseases, ICD‐10)^7^ and tumour morphology (coded using the second and third editions of the International Classification of Diseases for Oncology, ICD‐O).^8^, ^9^

### Outcomes

The outcome of interest was incident primary invasive colorectal cancer (ICD‐10 C18‐20). For analyses by tumour site, cancers were assigned to the following groups: colon, ICD‐10 C18.0‐18.9; colon right (proximal), C18.0 caecum to C18.4 transverse colon; colon left (distal), C18.5 splenic flexure to C18.7 sigmoid colon; rectum, C19‐C20. For analyses by morphological type, cancers were divided into five groups based on the WHO/IARC classification:^10^ (*i*) adenocarcinoma, ICD‐O code M8140/3 (and 12 related codes: Supporting Information Appendix Table 1); (*ii*) mucinous adenocarcinoma, ICD‐O M8480/3 (and three related codes); (*iii*) signet ring cell carcinoma, ICD‐O code M8490/3; (*iv*) neuroendocrine tumours, including carcinoid, ICD‐O codes M8246/3, M8240/3, M8243/3 and six related codes; and (*v*) other tumours: all remaining tumours, including other specified carcinoma, specified non‐carcinoma tumours, and cancers of unspecified morphology.

**Table 1:** Characteristics of women at recruitment, and follow‐up for incident colorectal cancer

	**All women in analysis**	**All women with colorectal cancer** **ICD‐10 C18‐C20**
	***n* = 1,310,390**	***n* = 18,518**
Age (yr)		
Mean (SD)	56.1 (4.6)	57.6 (4.7)
Socioeconomic status		
% Most deprived tertile	33.3	33.4
Height (cm)		
Mean (SD)	162.0 (6.7)	162.5 (6.7)
Body mass index (kg/m^2^)		
Mean (SD)	26.2 (4.7)	26.4(4.7)
Smoking		
% Current	20.6	19.9
Alcohol		
%15 + units/wk	5.0	5.7
Strenuous physical activity		
%1 + /wk	38.9	36.6
Age at menarche		
% 15+ yr	17.0	17.9
Parity		
% Nulliparous	10.8	11.7
*N* Pregnancies^a^		
%3+	37.0	39.0
Hysterectomy		
% Yes	25.0	24.3
Sterilisation		
% Yes	23.2	21.7
Age at menopause (yr)^b^		
Mean (SD)	47.5 (6.1)	49.4 (4.7)
Oral contraceptive use		
% Ever	33.2	29.9
Hormone therapy use^c^		
% Ever	50.5	46.9
Years of follow‐up		
Mean (SD)	13.8 (3.4)	8.8 (4.3)
Age at cancer diagnosis (yr)		
Mean (SD)	–	66.9 (6.2)

a In parous women.

b In never HT users.

c In postmenopausal women.

### Exposures

All exposure variables were measured at study recruitment. The 14 exposures studied were: socioeconomic status (tertiles of the area‐based Townsend deprivation index);^11^ height (<160 cm, 160–165 cm, 165 + cm); body mass index (BMI) (<25, 25–29 and 30+ kg/m^2^, calculated from reported height and weight); smoking status (never smoker, past smoker, current smoker <15 cigarettes/day, current smoker 15+ cigarettes/day; for some analyses, the two current smoking categories were combined); alcohol intake (0–2, 3–14.9, 15+ units/week, with one unit = 10 g alcohol); strenuous physical activity (rarely/never, up to once a week, more than once a week); use of hormone replacement therapy for menopause (HT) (never, ever, within postmenopausal women); age at menarche (<13, 13–14, 15+ years); parity (parous, nulliparous); number of full‐term pregnancies (1,2,3+, within parous women); age at menopause (<45, 45–49, 50+ years, within postmenopausal never users of HT); hysterectomy (yes, no), sterilisation (tubal ligation) (yes, no) and past use of oral contraceptives (OCs) (never, for <5 years, for 5+ years).

### Statistical analysis

Women were excluded from the analyses if they had been registered with any invasive cancer other than non‐melanoma skin cancer (ICD‐10 C44) prior to recruitment (*n* = 44,829). The remaining women contributed person‐years from the date of recruitment to the study until the date of registration for colorectal cancer, the date of death, or last date of follow‐up, whichever was soonest. Women were censored at diagnosis of any non‐colorectal cancer.

Cox (proportional hazards) regression models were used to estimate hazard ratios (referred to here as relative risks, RRs) of developing colorectal cancer in relation to the exposures of interest. Attained age was the underlying time variable. There was no evidence of significant violation of the proportional hazards assumption, as assessed by tests based on Schoenfeld residuals.

All analyses were stratified by geographical region (10 regions corresponding to the areas covered by the recruiting cancer registries), and mutually adjusted for other exposure variables as appropriate.

For adjustment and stratification variables, missing values (<6% for each variable) were assigned to a separate category. A sensitivity analysis was conducted excluding the 15% of women with any missing data on covariates within the model. A further sensitivity analysis was performed among the 1.2 million women in England, censoring at the date of first invitation to bowel cancer screening, (the NHS Bowel Cancer Screening Programme was introduced in England from 2006, and information on invitation date is available for study participants through linkage to screening data.^12^ No equivalent data are available for women recruited in Scotland.) Information on diet and on parental history of cancer is available for some 830,000 women in the study from the 3‐year re‐survey questionnaire. A sensitivity analysis was performed in these women, with follow‐up starting at the date of completion of the re‐survey questionnaire, and with additional adjustment for intake of red and processed meat (no red or processed meat, only red meat, red and processed meat) and dietary fibre (non‐starch polysaccharides [Englyst method], tertiles), and for history of colorectal cancer in mother and/or father (yes, no).

Tests of heterogeneity in the relationships between exposures and colorectal cancer risk by subsite and subtype were performed using a competing risks approach. We interpreted significance after correction of *p* values for multiple comparisons using the Holm‐Bonferroni method,^13^ describing corrected *p* < 0.05 as statistically significant. Analyses were performed in Stata‐14.^14^ Tests of statistical significance were two‐sided.

## Results

Overall 1,310,390 women without prior cancer, with a mean age at recruitment of 56.1 (SD 4.6) years, were included in the analyses. Women were followed for incident colorectal cancer over 18.1 million person‐years, with a mean duration of follow‐up of 13.8 (SD 3.4) years per woman. During this period, 18,518 incident primary invasive colorectal cancers were registered, with a mean age at diagnosis of 66.9 years (SD 6.2). By site, 12,761 cancers were in the colon (6,278 specified in the right colon and 5,269 in the left colon), and 5,757 in the rectum. The remaining 1,214 tumours were of overlapping or unknown site. By morphology, 15,543 cancers (83.9%) were reported as adenocarcinoma, 1,270 (6.8%) as mucinous tumours, 107 (0.6%) as signet ring cell tumours, and 234 (1.3%) as neuroendocrine tumours (predominantly carcinoid); the remaining 1,364 cancers were of other or unspecified histological type.

Table 1 shows characteristics of women in the analysis. Women diagnosed with colorectal cancer were somewhat less likely to smoke, undertake strenuous physical activity, or ever to have taken HT, and more likely to drink 15 or more units of alcohol a week. Characteristics did not vary much by cancer site, but there were some differences between women by tumour type (Supporting Information Appendix Table 2). Those diagnosed with signet ring or neuroendocrine tumours were more likely to be current smokers than those diagnosed with other tumour types; they tended also to be younger at recruitment and at cancer diagnosis.

**Table 2:** Distribution of colorectal cancers by site and by morphology

	**Adenocarcinoma**	**Mucinous**	**Signet ring**	**Neuroendocrine**	**Other and unspecified**	**Total**
**Colon, right**	**4,892**	**695**	**65**	**159**	**467**	**6,278**
Caecum	2,212	288	25	63	232	2,820
Appendix	39	85	8	87	14	233
Ascending colon	1,378	176	18	6	93	1,671
Hepatic flexure/transverse colon	1,263	146	14	3	128	1,554
**Colon, left**	**4,692**	**254**	**10**	**10**	**303**	**5,269**
Splenic flexure/descending colon	846	74	6	0	65	991
Sigmoid colon	3,846	180	4	10	238	4,278
**Colon, overlapping/unspecified**	**881**	**86**	**10**	**15**	**222**	**1,214**
**Rectum**	**5,078**	**235**	**22**	**50**	**372**	**5,757**
Recto‐sigmoid	1,228	47	8	2	84	1,369
Rectum	3,850	188	14	48	288	4,388
**Total**	**15,543**	**1,270**	**107**	**234**	**1,364**	**18,518**

Table 2 shows the distribution of colorectal cancers cross‐classified by site and type. While adenocarcinomas are approximately evenly distributed between right colon, left colon and rectum, mucinous, signet ring and (in particular) neuroendocrine tumours are predominantly found in the right colon.

Table 3 shows adjusted relative risks for incident colorectal cancer for the 14 exposures examined. Taking multiple testing into account, cancer risk was significantly increased (*p* < 0.05) in relation to taller height, greater BMI, smoking, higher alcohol intake, and in parous *versus* nulliparous women; and risk was significantly decreased in relation to higher levels of physical activity, and to ever use of hormone replacement therapy. No significant associations were seen for socioeconomic status or for the remaining reproductive factors examined (including number of children in parous women).

**Table 3:** Relative risks (RRs) and 95% confidence intervals (CIs) for incident colorectal cancer in relation to 14 socioeconomic and behavioural factors

	**Colorectal cancers,** **total = 18,518**	**RR (95% CI)**	***χ*** b **test for heterogeneity** (*p < 0.05 after Holm‐Bonferroni correction for multiple testing)
**Socioeconomic status**			
Least deprived	6,040	Reference	
Mid tertile	6,221	1.03 (0.99–1.07)	***χ*_**2**_^**2**^** *=* 3.36
Most deprived	6,146	1.03 (0.99–1.07)	
**Height (cm)**			
<160	5,438	Reference	
160–164.9	5,444	1.12 (1.08–1.16)	***χ*_**2**_^**2**^** = 156.4*
165+	7,333	1.25 (1.21–1.30)	
**Body mass index (kg/m^2^)**			
<25	7,751	Reference	
25–29.9	6,523	1.07 (1.04–1.11)	***χ*_**2**_^**2**^** = 27.5*
30+	3,262	1.11 (1.06–1.15)	
**Smoking**			
Never	8,610	Reference	
Past	5,385	1.15 (1.11–1.19)	***χ*_**3**_** b = 85.5*
Current <15/d	1,797	1.13 (1.07–1.19)	
Current 15+/d	1,668	1.19 (1.13–1.26)	
**Alcohol (units/wk)**			
0–2	10,947	Reference	
3–14.9	6,389	1.04 (1.01–1.08)	***χ*_**2**_^**2**^** = 44.9*
15+	1,049	1.25 (1.17–1.33)	
**Strenuous exercise**			
Rarely/never	9,112	Reference	
Once per week	5,222	0.95 (0.91–0.98)	***χ*_**2**_^**2**^** = 28.4*
>Once per week	3,498	0.90 (0.87–0.94)	
**Age at menarche (yr)**			
<13	6,833	Reference	
13–14	8,063	0.96 (0.93–0.99)	***χ*_**2**_^**2**^** = 7.07
15+	3,238	0.98 (0.93–1.02)	
Parity			
Nulliparous	2,166	Reference	***χ*_**1**_** b = 17.36*
Parous	16,310	0.91 (0.87–0.95)	
**Births (in parous women)**			
1	2,612	Reference	
2	7,341	0.97 (0.92–1.01)	***χ*_**2**_^**2**^** = 3.19
3+	6,357	0.99 (0.95–1.04)	
**Hysterectomy**			
No	13,916	Reference	***χ*_**1**_** b = 4.72
Yes	4,461	0.96 (0.92–1.00)	
**Sterilisation**			
No	14,043	Reference	***χ*_**1**_** b = 0.15
Yes	3,895	0.99 (0.96–1.03)	
**Age at menopause** a **(yr)**			
<45	900	Reference	
45–49	1,949	0.93 (0.86–1.01)	***χ*_**2**_^**2**^** = 3.23
50+	4,098	0.97 (0.90–1.04)	
**Oral contraceptive use (yr)**			
Never	8,422	Reference	
<5	4,403	0.98 (0.94–1.02)	***χ*_**2**_^**2**^** = 1.28
5+	5,479	0.99 (0.95–1.02)	
**Hormone therapy use** b			
Never	7,134	Reference	***χ*_**1**_** b = 8.87*
Ever	4,130	0.94 (0.90–0.98)	

a In postmenopausal never HT users.

b In postmenopausal women.

**Figure 1:**
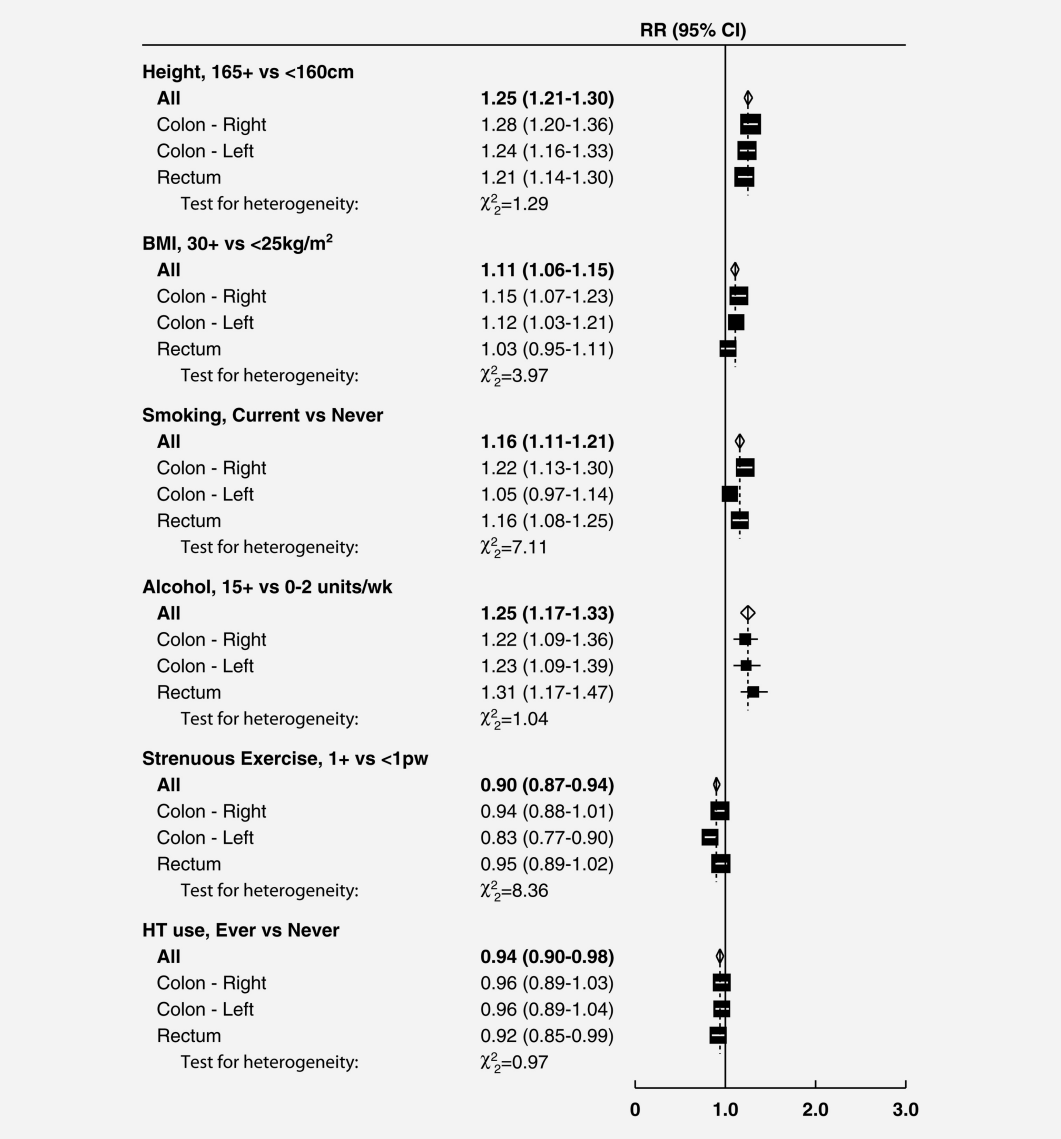
Relative risks (RRs) and 95% confidence intervals (CIs) for incident colorectal cancer in relation to selected risk factors, by anatomic site. All tests for heterogeneity are non‐significant (*p* > 0.05) after Holm‐Bonferroni correction for multiple testing BMI, body mass index; HT, hormone therapy for menopause; pw, per week

**Figure 2:**
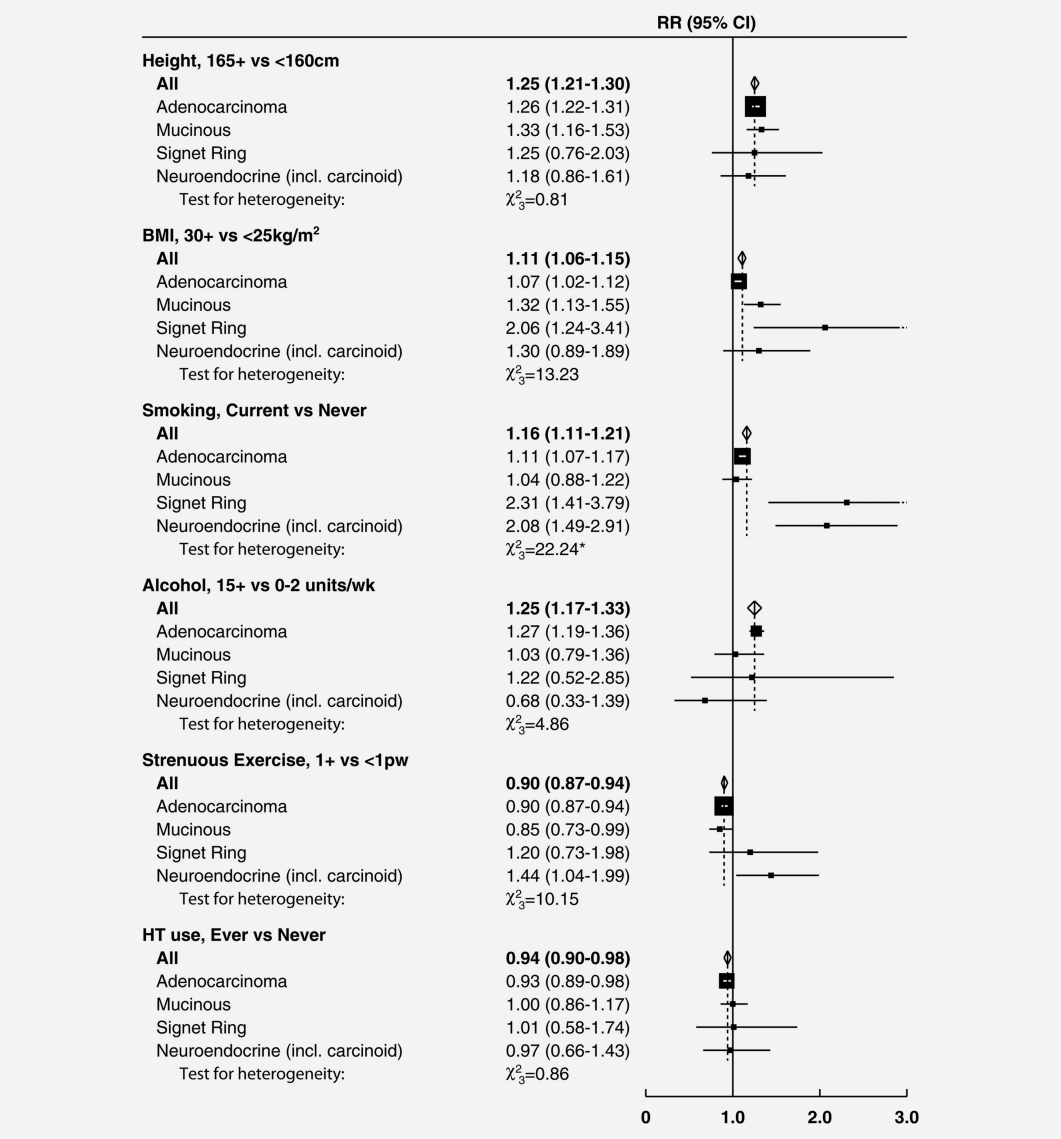
Relative risks (RRs) and 95% confidence intervals (CIs) for incident colorectal cancer in relation to selected risk factors, by morphology. Tests for heterogeneity are non‐significant (*p* > 0.05) after Holm‐Bonferroni correction for multiple testing, except where indicated by *p > 0.05. BMI, body mass index; HT, hormone therapy for menopause; pw, per week

Figures 1 and 2 show, respectively, results by site and by tumour morphology for six factors most strongly associated with risk of colorectal cancer. Details of the analyses for all exposures by site and by morphology are shown in the Supporting Information Appendix Tables 3 and 4. Results for parity *versus* nulliparity are not included in the figures (but are given in the Supporting Information Appendix) as the inconsistency with associations by number of births among parous women suggests that this finding may well be due to chance.

There was no significant heterogeneity by tumour location for any of the 14 factors examined (Fig. 1 and Supporting Information Appendix Table 3). Associations for smoking varied substantially, however, by tumour morphology (Fig. 2). For two rare subtypes of colorectal cancer, signet ring cell and neuroendocrine tumours, risks were doubled in current *versus* never smokers (RRs = 2.31, 95% CI 1.41–3.79 and 2.08, 1.49–2.91 respectively); but for the most common subtype, adenocarcinoma, any association with smoking was weak (1.11, 1.07–1.17). In the more detailed analyses, risks for both signet ring cell and neuroendocrine tumours were substantially higher for current smokers of 15+ cigarettes per day (RRs around 3) than for current smokers of <15 cigarettes per day (RRs around 1.6), with *p* < 0.001 for heterogeneity by tumour morphology (Supporting Information Appendix Table 4). Obesity (BMI 30+ *vs*. <25 kg/m^2^) was more strongly associated with signet ring cell (RR 2.06, 1.24–3.41), mucinous (1.32, 1.13–1.55) and neuroendocrine tumours (1.30, 0.89–1.89) than with adenocarcinoma (1.07, 1.02–1.12); these differences were of borderline statistical significance (corrected *p* = 0.055). There was no significant heterogeneity by tumour morphology for height, alcohol intake, use of HT, physical activity or parity (Fig. 2, Supporting Information Appendix Table 4); nor for the other seven risk factors examined (Supporting Information Appendix Table 4).

Results from the three sensitivity analyses (restricting to women with no missing information on covariates; censoring at first invitation to bowel cancer screening; with additional adjustment for dietary factors and for parental history of colorectal cancer) did not differ substantially from those reported above (Supporting Information Appendix Table 5).

## Discussion

In this large cohort of UK women, overall risk of incident invasive colorectal cancer was positively associated with height, body mass index, both past and current smoking, and alcohol intake; and inversely associated with physical activity and with use of hormone therapy for the menopause. The relative risk estimates for colorectal cancer were small, generally in the range 0.9 to 1.2. Colorectal cancer risk was lower in parous than in nulliparous women, but there was no trend in risk by number of births among parous women, and no associations were found with other reproductive factors, or with socioeconomic status. For smoking there was substantial heterogeneity across tumour morphological types; strong associations, with relative risks around 2 or greater were seen in current smokers for both signet ring cell and neuroendocrine tumours. Obese women were at similarly increased relative risk for signet ring cell tumours. For adenocarcinoma, the large majority of colorectal cancers in the cohort, the associations with BMI and with smoking were weak, with relative risks around 1.1. No significant differences in risk by tumour location were seen for any of the 14 factors examined.

This large prospective study, with 18,600 incident colorectal cancers, provides an opportunity to study risk factor associations in detail, taking account of potential confounding factors. In particular we were able to compare directly associations both by tumour site and by morphological type. For colorectal cancer overall, our results are consistent with past findings of small increases in risk of incident cancer in relation to taller height, adiposity, both past and current smoking, and moderately high alcohol intake; and of small decreases in risk associated with physical exercise, and with use of hormone therapy for menopause. Reproductive factors were in general not associated with risk of colorectal cancer; the lower risk in parous women is not accompanied by evidence for a trend in risk by number of births, and may be due to chance.

Existing evidence on risk factor associations by site of colorectal cancer is inconsistent.^15^, ^16^, ^17^ It has been suggested that risks may differ between colon and rectal cancers, or between right‐sided and left‐sided colon (or colorectal) cancers,^18^, ^19^ but results have been varied and interpretation often hindered by lack of statistical power to test reliably for differences between sites. We found little to suggest differences by tumour site for the 14 risk factors examined.

All the associations between smoking and cancer risk by site were modest. By contrast, the associations we found between smoking and risk of signet ring cell and neuroendocrine tumours are stronger and the results suggest a trend in risk with amount smoked (Supporting Information Appendix Table 3). While there is limited understanding of the relationship between morphological and molecular subtypes of colorectal cancer,^20^ our results on smoking appear consistent with recent evidence from molecular epidemiological studies, which have suggested a stronger association between smoking and risk of colorectal tumours^21^, ^22^, ^23^, ^24^, ^25^ and polyps^26^, ^27^ thought to arise from non‐conventional pathways (such as the serrated neoplasia pathway)^2^ than with adenocarcinomas arising from the conventional pathway typified by chromosomal instability. Less is known of possible differential risks for colorectal cancer by molecular (or morphological) type for other exposures. Our findings suggest that obesity may also be a stronger risk factor for the less common tumour types, in particular signet ring cell tumours, than for adenocarcinoma.

Strengths of this large study include prospectively‐collected information on potential risk and confounding factors, and virtually complete follow‐up through linkage to routinely‐collected, reliable, cancer registration data, giving sufficient power to compare risk associations directly both by site and by morphological type. The majority of colorectal cancers in this study were diagnosed in women who had not had access to bowel cancer screening, and results remained similar when women invited for screening were excluded from the analyses; screening acceptance has been shown in this cohort to be related to individual characteristics, such as smoking and BMI,^12^ and differential access to diagnosis could potentially bias studies of risk factors in screened populations. A limitation is that there was no independent assessment of the morphological classification of tumours, but misclassification (assumed to be unrelated to exposure status) would be likely to attenuate, rather than create, differences in risk associations by type. Even in this large study, we had limited numbers of tumours of rarer subtypes. The extent to which our findings are generalisable to men, and to other populations is not known.

We have shown in this large prospective study in UK women that associations of colorectal cancer risk with major lifestyle risk factors do not vary greatly by tumour site. There are however some substantial differences by morphological type, smoking in particular being associated with two to threefold increased risk of signet ring call carcinoma and of neuroendocrine tumours, but only weakly associated with risk of adenocarcinoma.

**Abbreviations**
BMI: body mass index
CI: confidence interval
HT: hormone therapy for menopause
ICD‐10: Tenth revision of the International Classification of Diseases
ICD‐O: International Classification of Diseases for Oncology
NHS: National Health Service
OC: oral contraceptive
RR: relative risk
SD: standard deviation

## Supporting information

Supporting Information
