# Supplementary material for: Heterogeneity of colorectal cancer risk by tumour characteristics: Large prospective study of UK women
Source: Int J Cancer. 2017 Jan 18;140(5):1082–90. doi: 10.1002/ijc.30527 (PMC5347941; doi:10.1002/ijc.30527)
Supplement: Supplementary file 1 — Supporting Information [file IJC-140-1082-s001.docx]

**Appendix Table 1: Colorectal cancers, grouped by ICD-0 morphology code**

| Group | ICD-O code | n |
| --- | --- | --- |
| **Adenocarcinoma**  Adenocarcinoma NOS  Adenocarcinoma, intestinal type  Adenocarcinoma in adenomatous polyp  Tubular adenocarcinoma  Adenocarcinoma in villous adenoma  Villous adenocarcinoma  Adenocarcinoma in tubulovillous adenoma  Papillary adenocarcinoma NOS  Other adenocarcinoma  Adenocarcinoma in multiple  adenomatous polyps  Scirrhous adenocarcinoma  Solid adenocarcinoma NOS  Adenocarcinoma, mixed subtypes  Superficial spreading adenocarcinoma | M8140/3  M8144/3  M8210/3  M8211/3  M8261/3  M8262/3  M8263/3  M8260/3  M8221/3  M8141/3  M8230/3  M8255/3  M8143/3 | **Total: 15 543**  14 053  159  348  47  140  17  763  8  8 |
| **Mucinous adenocarcinoma**  Mucinous adenocarcinoma  Mucin-producing adenocarcinoma  Mucinous cystadenocarcinoma | M8480/3  M8481/3  M8470/3  M8472/3 | **Total: 1270**  1 066  200  4 |
| **Signet Ring cell carcinoma** | M8490/3 | **Total: 107** |
| **Neuroendocrine tumours**  Neuroendocrine NOS  Carcinoid NOS  Goblet cell carcinoid  Other neuroendocrine  Carcinoid, argentaffin  Composite carcinoid  Adenocarcinoid  Large cell carcinoma, NOS  Small cell carcinoma, NOS  Large cell neuroendocrine carcinoma | M8246/3  M8240/3  M8243/3  M8241/3  M8244/3  M8245/3  M8012/3  M8041/3  M8013/3 | **Total: 234**  53  123  40  18 |
| **Other tumours** |  | **Total: 1364** |
| Malignant neoplasm NOS  Epithelial neoplasms NOS  Other epithelial NOS Tumorlet  Epithelial  Carcinoma undifferentiated  Carcinoma anaplastic NOS | M8000/3  M8010/3  M8040/3  M8046/3  M8020/3  M8021/3 | 604  633  11 |
| Squamous cell carcinoma  Other epithelial  Medullary carcinoma  Adenosquamous carcinoma  Acinar cell carcinoma  Basaloid carcinoma  Adenocarcinoma with squamous metaplasia  Cloacogenic carcinoma  Mixed cell adenocarcinoma  Cystadenocarcinoma  Complex epithelial/adenocarcinoma | M8070/3  M8071/3  M8072/3  M8510/3  M8560/3  M8550/3  M8123/3  M8570/3  M8124/3  M8323/3  M8440/3  M8574/3 | 75  28 |
| Other, non-epithelial  Leiomyosarcoma  Sarcoma nos  Sarcoma spindle cell  Fibrosarcoma  Liposarcoma  Sarcoma  Adenosarcoma  Malignant melanoma | M8890/3  M8800/3  M8801/3  M8810/3  M8851/3  M8930/3  M8933/3  M8720/3  M8140/5 | 13 |
| **TOTAL** |  | **18 518** |

Appendix Table 2: Characteristics and follow-up of women with colorectal cancer, by tumour location and tumour morphology

|  | **All CRC**  **C18-20** | **Colon All**  **C18.0-18.9** | **Colon Right**  **C18.0-18.4** | **Colon Left**  **C18.5-18.7** | **Rectum**  **C19-20** | **Adeno-carcinoma** | **Mucinous** | **Signet Ring** | **Neuro-endocrine** |
| --- | --- | --- | --- | --- | --- | --- | --- | --- | --- |
| N cases | **18,518** | **12,761** | **6,278** | **5,269** | **5,757** | **15,543** | **1,270** | **107** | **234** |
| Age, years  mean, (SD) | 57.6 (4.7) | 57.8 (4.7) | 57.9(4.7) | 57.6 (4.7) | 57.3 (4.7) | 57.6 ( 4.7) | 58.1 (4.7) | 56.5 (5.0) | 56.2 ( 4.5) |
| SES, % most deprived tertile | 33.4 | 33.2 | 33.9 | 32.2 | 33.8 | 33.4 | 32.5 | 36.5 | 32.9 |
| Height, cm mean, (SD) | 162.5 (6.7) | 162.6 (6.8) | 162.5 (6.8) | 162.5 (6.9) | 162.4 (6.6) | 162.5 (6.7) | 162.6 (6.6) | 162.0 (6.2) | 162.6 (6.8) |
| BMI, kg/m^2^ mean, (SD) | 26.4 (4.7) | 26.5 (4.7) | 26.5 (4.7) | 26.5 (4.7) | 26.2 (4.6) | 26.3 (4.6) | 26.7 (4.7) | 27.2 (5.2) | 26.5 (4.2) |
| Smoking % current | 19.9 | 19.7 | 20.5 | 18.1 | 20.2 | 19.3 | 18.3 | 30.0 | 29.8 |
| Alcohol %15+units/week | 5.7 | 5.6 | 5.6 | 5.6 | 6.0 | 5.8 | 4.4 | 5.6 | 3.4 |
| Physical activity % 1+/week | 36.6 | 36.2 | 36.5 | 36.1 | 37.4 | 36.9 | 34.0 | 40.8 | 41.2 |
| Age at menarche, % 15+ years | 17.9 | 17.8 | 17.3 | 18.4 | 17.9 | 17.9 | 18.3 | 16.2 | 13.8 |
| Parity, % nulliparous | 11.7 | 11.7 | 11.6 | 11.6 | 11.8 | 11.6 | 10.4 | 8.4 | 10.3 |
| N Pregnancies*,  % 3+ | 39.0 | 39.3 | 39.7 | 39.0 | 38.2 | 38.7 | 40.3 | 34.7 | 36.2 |
| Hysterectomy, % Yes | 24.3 | 24.5 | 25.4 | 23.5 | 23.7 | 24.1 | 24.8 | 24.3 | 29.2 |
| Sterilisation, % Yes | 21.7 | 21.8 | 21.6 | 22.1 | 21.6 | 21.9 | 21.8 | 25.7 | 18.6 |
| Age at menopause,yrs^+^  Mean (SD) | 49.4 (4.7) | 49.4 (4.7) | 49.3 (4.7) | 49.5 (4.6) | 49.4 (4.6) | 49.4 (4.6) | 49.5 (4.6) | 48.6 (4.4) | 48.8 (5.1) |
| OC use, % ever | 29.9 | 29.9 | 30.0 | 29.5 | 30.0 | 30.1 | 28.4% | 23.4% | 36.2 |
| HT use, % ever | 46.9 | 47.1 | 47.4 | 46.9 | 46.3 | 47.0 | 46.3 | 44.9 | 52.6 |
|  |  |  |  |  |  |  |  |  |  |
| Follow-up, years  mean, (SD) | 8.8 (4.3) | 9.0 (4.3) | 9.5 (4.1) | 8.7 (4.3) | 8.4 (4.2) | 8.7 (4.3) | 8.6 (4.1) | 9.2 (4.3) | 9.1 (4.4) |
| Age at cancer diagnosis, years  mean, (SD) | 66.9 (6.2) | 67.2 (6.2) | 67.9 (6.1) | 66.7 (6.1) | 66.2 (6.2) | 66.7 (6.2) | 67.2 (6.0) | 66.1 (5.4) | 65.8 (5.9) |

CRC= colorectal cancer; SES= socioeconomic status; OC= oral contraceptive; HT= hormone therapy for menopause (in postmenopausal women)

* = in parous women ^+^= in never HT users

**Appendix Table 3: Relative risks (RRs) and 95% confidence intervals (CIs) for incident colorectal cancer in relation to 14 risk factors, by tumour location**

|  | **Colon All**    **C18.0-18.9**  **n= 12,761**  RR,95%CI | **Colon Right**  **C18.0-18.4**  **n= 6278**  RR,95%CI | **Colon Left**  **C18.5-18.7**  **n=5269**  RR,95%CI | **Rectum**  **C19-20**  **n=5757**  RR,95%CI | **Χ^2^ test for heterogeneity by site: colon left, colon right, rectum.**  **(* indicates p<0.05 after Holm-Bonferroni correction for multiple testing)** |
| --- | --- | --- | --- | --- | --- |
| **Socioeconomic status**  Least deprived  Mid tertile  Most deprived | *reference*  1.03 (0.99,1.07)  1.02 (0.98,1.07) | *reference*  1.04 (0.98,1.10)  1.06 (0.99,1.13) | *reference*  1.03 (0.96,1.10)  0.98 (0.91,1.05) | *reference*  1.03 (0.97,1.10)  1.05 (0.98,1.12) | **Χ_2_^2^ = 2.85** |
| **Height**  <160 cm  160-164.9 cm  165+ cm | *reference*  1.10 (1.05,1.15)  1.27 (1.22,1.33) | *reference*  1.10 (1.03,1.17)  1.28 (1.20,1.36) | *reference*  1.08 (1.01,1.16)  1.24 (1.16,1.33) | *reference*  1.17 (1.09,1.25)  1.21 (1.14,1.30) | **Χ_2_^2^ = 1.29** |
| **Body Mass Index, kg/m^2^**  <25  25-29.9  30+ | *reference*  1.08 (1.04,1.13)  1.14 (1.08,1.20) | *reference*  1.07 (1.01,1.14)  1.15 (1.07,1.23) | *reference*  1.11 (1.04,1.18)  1.12 (1.03,1.21) | *reference*  1.05 (0.99,1.11)  1.03 (0.95,1.11) | **Χ_2_^2^ = 3.97** |
| **Smoking, category** Never  Past  Current | *reference*  1.14 (1.09,1.19)  1.16 1.10,1.22) | *reference*  1.06 (0.99,1.12)  1.22 (1.13,1.30) | *reference*  1.22 (1.14,1.30)  1.05 (0.97,1.14) | *reference*  1.17 (1.10,1.25)  1.16 (1.08,1.25) | **Χ_2_^2^ = 7.11** |
| **Smoking, exposure level** Never  Current <15/day  Current 15+/day | *reference*  1.12 (1.05,1.19)  1.21 (1.13,1.29) | *reference*  1.12 (1.03,1.23)  1.33 (1.22,1.46) | *reference*  1.09 (0.99,1.21)  1.01 (0.91,1.12) | *reference*  1.16 (1.06,1.28)  1.16 (1.05,1.28) | **Χ_2_^2^ =14.92*** |
| **Alcohol, units/ week**  0-2  3-14.9  15+ | *reference*  1.02 (0.98,1.06)  1.22 (1.13,1.32) | *reference*  0.97 (0.91,1.03)  1.22 (1.09,1.36) | *reference*  1.07 (1.01,1.13)  1.23 (1.09,1.39) | *reference*  1.09 (1.03,1.16)  1.31 (1.17,1.47) | **Χ_2_^2^= 1.04** |
| **Strenuous exercise**  Rarely/never  Up to once per week  > 1 per week | *reference*  0.94 (0.90,0.98)  0.88 (0.84,0.92) | *reference*  0.94 (0.89,1.00)  0.94 (0.88,1.01) | *reference*  0.93 (0.88,1.00)  0.83 (0.77,0.90) | *reference*  0.96 (0.90,1.02)  0.95 (0.89,1.02) | **Χ_2_^2^ = 8.36** |
| **Age at menarche, years**  <13  13-14  15+ | *reference*  0.96 (0.93,1.00)  0.97 (0.93,1.02) | *reference*  0.95 (0.90,1.00)  0.92 (0.85,0.99) | *reference*  0.96 (0.91,1.03)  1.02 (0.95,1.11) | *reference*  0.94 (0.89,1.00)  0.98 (0.91,1.06) | **Χ_2_^2^ = 4.14** |
| **Parity**  Nulliparous  Parous | *reference*  0.91 (0.86,0.96) | *reference*  0.91 (0.84,0.98) | *reference*  0.91 (0.84,1.00) | *reference*  0.91 (0.83,0.98) | **Χ_2_^2^ = 0.03** |
| **Births (in parous women)**  1  2  3+ | *reference*  0.98 (0.93,1.03)  1.01 (0.95,1.06) | *reference*  1.00 (0.92,1.08)  1.02 (0.94,1.10) | *reference*  0.97 (0.89,1.06)  1.00 (0.92,1.10) | *reference*  0.94 (0.86,1.01)  0.96 (0.88,1.04) | **Χ_2_^2^ = 3.19** |
| **Hysterectomy**  No  Yes | *reference*  0.96 (0.91,1.01) | *reference*  0.98 (0.91,1.05) | *reference*  0.92 (0.85,0.99) | *reference*  0.95 (0.89,1.03) | **Χ_2_^2^ = 1.49** |
| **Sterilisation**  No  Yes | *reference*  1.00 (0.95,1.04) | *reference*  0.99 (0.93,1.06) | *reference*  1.02 (0.95,1.09) | *reference*  0.98 (0.92,1.05) | **Χ_2_^2^ = 0.65** |
| **Age at menopause^^^, years**  <45  45-49  50+ | *reference*  0.90 (0.82,0.99)  0.95 (0.87,1.04) | *reference*  0.89 (0.78,1.02)  0.92 (0.81,1.04) | *reference*  0.93 (0.80,1.08)  0.97 (0.85,1.12) | *reference*  1.01 (0.87,1.17)  1.02 (0.89,1.16) | **Χ_2_^2^ = 0.86** |
| **Oral Contraceptive use**  Never  <5 years  5+years | *reference*  0.99 (0.95,1.04)  1.01 (0.97,1.05) | *reference*  1.05 (0.98,1.12)  1.08 (1.01,1.15) | *reference*  0.97 (0.90,1.04)  0.95 (0.88,1.01) | *reference*  0.95 (0.89,1.01)  0.94 (0.88,1.00) | **Χ_2_^2^ = 11.17*** |
| **Hormone therapy use**  Never  Ever | *reference*  0.95 (0.90,1.00) NS | *reference*  0.96 (0.89,1.03) NS | *reference*  0.96 (0.89,1.04) | *reference*  0.92 (0.85,0.99) | **Χ_2_^2^ = 0.97** |

**^** among never users of HT; ^+^ among postmenopausal women

**Appendix Table 4: Relative risks (RRs) and 95% confidence intervals (CIs) for incident colorectal cancer in relation to 14 risk factors, by tumour morphology**

|  | **Adeno-carcinoma**  **n= 15,543**  RR,95%CI | **Mucinous**  **n= 1270**  RR,95%CI | **Signet Ring**  **n=107**  RR,95%CI | **Neuro-endocrine**  **n= 234**  RR,95%CI | **Χ^2^ test for heterogeneity by morphology (* indicates p<0.05 after Holm-Bonferroni correction for multiple testing)** |
| --- | --- | --- | --- | --- | --- |
| **Socioeconomic status**  Least deprived  Mid tertile  Most deprived | *reference*  1.02 (0.98,1.06)  1.03 (0.99,1.07) | *reference*  1.07 (0.93,1.22)  1.00 (0.87,1.16) | *reference*  0.88 (0.55,1.42)  0.96 (0.60,1.54) | *reference*  1.08 (0.79,1.48)  0.96 (0.69,1.34) | **Χ_3_^2^ = 0.38** |
| **Height**  <160 cm  160-164.9 cm  165+ cm | *reference*  1.13 (1.08,1.18)  1.26 (1.22,1.31) | *reference*  1.13 (0.97,1.30)  1.33 (1.16,1.53) | *reference*  1.53 (0.95,2.46)  1.25 (0.76,2.03) | *reference*  1.04 (0.75,1.45)  1.18 (0.86,1.61) | **Χ_3_^2^ = 0.81** |
| **Body Mass Index, kg/m^2^**  <25  25-29.9  30+ | *reference*  1.06 (1.03,1.10)  1.07 (1.02,1.12) | *reference*  1.17 (1.03,1.33)  1.32 (1.13,1.55) | *reference*  1.07 (0.67,1.72)  2.06 (1.24,3.41) | *reference*  1.31 (0.98,1.77)  1.30 (0.89,1.89) | **Χ_3_^2^ = 13.23** |
| **Smoking, category** Never  Past  Current | *reference*  1.16 (1.12,1.21)  1.11 (1.07-1.17) | *reference*  1.04 (0.91,1.19)  1.04 (0.88-1.22) | *reference*  1.39 (0.86,2.26)  2.31 (1.41-3.79) | *reference*  1.24 (0.90,1.72)  2.08 (1.49,2.91) | **Χ_3_^2^ = 22.24*** |
| **Smoking, amount** Never  Current <15/day  Current 15+/day | *reference*  1.09 (1.03,1.16)  1.14 (1.07,1.21) | *reference*  1.15 (0.95,1.39)  0.90 (0.72,1.14) | *reference*  1.61 (0.82,3.18)  3.14 (1.77,5.57) | *reference*  1.64 (1.06,2.54)  2.61 (1.76,3.88) | **Χ_3_^2^ = 32.45*** |
| **Alcohol, units/ week**  0-2  3-14.9  15+ | *reference*  1.06 (1.02,1.09)  1.27 (1.19,1.36) | *reference*  1.06 (0.94,1.20)  1.03 (0.79,1.36) | *reference*  0.97 (0.64,1.49)  1.22 (0.52,2.85) | *reference*  1.09 (0.83,1.43)  0.68 (0.33,1.39) | **Χ_3_^2^ = 4.86** |
| **Strenuous exercise**  Rarely/never  Up to once per week  > 1 per week | *reference*  0.95 (0.91,0.98)  0.90 (0.87,0.94) | *reference*  0.90 (0.78,1.02)  0.85 (0.73,0.99) | *reference*  0.95 (0.59,1.53)  1.20 (0.73,1.98) | *reference*  1.00 (0.73,1.38)  1.44 (1.04,1.99) | **Χ_3_^2^ = 10.15** |
| **Age at menarche, years**  <13  13-14  15+ | *reference*  0.94 (0.91,0.98)  0.97 (0.92,1.01) | *reference*  1.04 (0.91,1.17)  1.06 (0.90,1.24) | *reference*  1.37 (0.89,2.12)  1.10 (0.61,1.98) | *reference*  0.75 (0.56,1.00)  0.65 (0.43,0.98) | **Χ_3_^2^ = 4.96** |
| **Parity**  Nulliparous  Parous | *reference*  0.91 (0.87,0.96) | *reference*  1.04 (0.87,1.25) | *reference*  1.29 (0.64,2.58) | *reference*  1.03 (0.67,1.59) | **Χ_3_^2^ = 2.93** |
| **Births (in parous women)**  1  2  3+ | *reference*  0.96 (0.91,1.00)  0.97 (0.93,1.03) | *reference*  1.01 (0.85,1.21)  1.07 (0.90,1.28) | *reference*  0.98 (0.56,1.71)  0.76 (0.42,1.37) | *reference*  1.07 (0.72,1.59)  1.01 (0.67,1.53) | **Χ_3_^2^ = 1.94** |
| **Hysterectomy**  No  Yes | *reference*  0.95 (0.90,0.99) | *reference*  0.97 (0.83,1.13) | *reference*  1.05 (0.61,1.82) | *reference*  1.15 (0.82,1.61) | **Χ_3_^2^ = 5.89** |
| **Sterilisation**  No  Yes | *reference*  1.00 (0.96,1.04) | *reference*  0.99 (0.86,1.14) | *reference*  1.15 (0.72,1.84) | *reference*  0.70 (0.50,0.99) | **Χ_3_^2^ = 5.00** |
| **Age at menopause^^^, years**  <45  45-49  50+ | *reference*  0.94 (0.86,1.02)  0.98 (0.91,1.07) | *reference*  1.13 (0.82,1.54)  1.07 (0.79,1.43) | *reference*  0.71 (0.26,1.94)  0.58 (0.23,1.48) | *reference*  0.58 (0.29,1.15)  0.61 (0.33,1.15) | **Χ_3_^2^ = 2.67** |
| **Oral Contraceptive use**  Never  <5 years  5+years | *reference*  0.98 (0.94,1.02)  0.99 (0.95,1.03) | *reference*  0.97 (0.84,1.11)  0.95 (0.83,1.09) | *reference*  0.84 (0.52,1.34)  0.60 (0.37,0.99) | *reference*  1.24 (0.88,1.74)  1.37 (0.99,1.88) | **Χ_3_^2^ = 8.12** |
| **Hormone therapy use^+^**  Never  Ever | *reference*  0.93 (0.89,0.98) | *reference*  1.00 (0.86,1.17) | *reference*  1.01 (0.58,1.74) | *reference*  0.97 (0.66,1.43) | **Χ_3_^2^ = 0.86** |

**^** among never users of HT; ^+^ among postmenopausal women

**Appendix Table 5: Relative risks (RRs) and 95% confidence intervals (CIs) for incident colorectal cancer in relation to 14 risk factors: sensitivity analyses (see Methods for details)**

| **Category** | **Main Analysis**  **RR (95% CI)** | **Additionally adjusted by diet and family history**  **RR (95% CI)** | **Censored at invitation to bowel Screening**  **RR (95% CI)** | **Restricted to women with no missing values**  **RR (95% CI)** |
| --- | --- | --- | --- | --- |
| **Socioeconomic status** Least deprived  Mid tertile  Most deprived | *reference*  1.03 (0.99,1.07)  1.03 (0.99,1.07) | *reference*  1.04 (0.99,1.09)  1.02 (0.97,1.08) | *reference*  1.04 (1.00,1.09)  1.05 (1.01,1.10) | *reference*  1.04 (0.99,1.08)  1.03 (0.98,1.08) |
| **Height** <160 cm  160-164.9 cm  165+ cm | *reference*  1.12 (1.08,1.16)  1.25 (1.21,1.30) | *reference*  1.10 (1.04,1.16)  1.25 (1.19,1.31) | *reference*  1.12 (1.07,1.17)  1.28 (1.23,1.34) | *reference*  1.13 (1.08,1.19)  1.29 (1.24,1.35) |
| **Body Mass Index, kg/m2** <25  25-29.9  30+ | *reference*  1.07 (1.04,1.11)  1.11 (1.06,1.15) | *reference*  1.08 (1.03,1.12)  1.12 (1.05,1.18) | *reference*  1.05 (1.01,1.09)  1.12 (1.07,1.18) | *reference*  1.08 (1.04,1.12)  1.10 (1.04,1.15) |
| **Smoking** Never  Past  Current <15/day  Current 15+/day | *reference*  1.15 (1.11,1.19)  1.13 (1.07,1.19)  1.19 (1.13,1.26) | *reference*  1.13 (1.08,1.18)  1.15 (1.07,1.24)  1.18 (1.09,1.28) | *reference*  1.14 (1.10,1.19)  1.09 (1.03,1.16)  1.13 (1.06,1.21) | *reference*  1.15 (1.11,1.20)  1.11 (1.04,1.18)  1.20 (1.13,1.28) |
| **Alcohol** 0-2 units pw  3-14.9 units pw  15+ units pw | *reference*  1.04 (1.01,1.08)  1.25 (1.17,1.33) | *reference*  1.03 (0.99,1.08)  1.28 (1.18,1.39) | *reference*  1.04 (1.00,1.08)  1.21 (1.12,1.31) | *reference*  1.04 (1.00,1.08)  1.26 (1.16,1.36) |
| **Strenuous exercise** Rarely/never  Up to 1 pw  > 1 pw | *reference*  0.95 (0.91,0.98)  0.90 (0.87,0.94) | *reference*  0.97 (0.92,1.01)  0.94 (0.89,0.99) | *reference*  0.93 (0.89,0.97)  0.90 (0.86,0.94) | *reference*  0.93 (0.89,0.97)  0.89 (0.85,0.94) |
| **Hormone Therapy use** Never  (postmenopausal women) Ever | *reference*  0.94 (0.90,0.98) | *reference*  0.96 (0.91,1.02) | *reference*  0.94 (0.90,0.99) | *reference*  0.94 (0.90,0.99) |
| **Age of Menarche (years)** <13  13-14  15+ | *reference*  0.96 (0.93,0.99)  0.98 (0.93,1.02) | *reference*  0.94 (0.90,0.99)  0.96 (0.91,1.02) | *reference*  0.96 (0.92,1.00)  0.98 (0.94,1.03) | *reference*  0.95 (0.91,0.99)  0.98 (0.93,1.03) |
| **Hysterectomy** No  Yes | *reference*  0.96 (0.92,1.00) | *reference*  0.94 (0.89,1.00) | *reference*  0.94 (0.90,0.99) | *reference*  0.98 (0.93,1.03) |
| **Sterilisation** No  Yes | *reference*  0.99 (0.96,1.03) | *reference*  0.97 (0.92,1.02) | *reference*  1.01 (0.97,1.06) | *reference*  0.99 (0.94,1.03) |
| **Oral Contraceptive use** Never  <5 years  5+years | *reference*  0.98 (0.94,1.02)  0.99 (0.95,1.02) | *reference*  0.98 (0.93,1.03)  0.99 (0.94,1.04) | *reference*  0.95 (0.91,1.00)  0.96 (0.92,1.01) | *reference*  0.97 (0.93,1.01)  1.00 (0.96,1.04) |
| **Parity** Nulliparous  Parous | *reference*  0.91 (0.87,0.95) | *reference*  0.92 (0.86,0.98) | *reference*  0.89 (0.85,0.94) | *reference*  0.91 (0.86,0.96) |
| **Births (in parous)** 1  2  3+ | *reference*  0.97 (0.92,1.01)  0.99 (0.95,1.04) | *reference*  0.95 (0.89,1.00)  0.97 (0.91,1.03) | *reference*  0.97 (0.92,1.03)  1.02 (0.97,1.08) | *reference*  0.96 (0.91,1.01)  0.97 (0.92,1.03) |
| **Age at Menopause, years** <45  (never HT users) 45-49  50+ | *reference*  0.93 (0.86,1.01)  0.97 (0.90,1.04) | *reference*  0.95 (0.85,1.06)  0.98 (0.88,1.09) | *reference*  0.91 (0.83,0.99)  0.95 (0.87,1.03) | *reference*  0.95 (0.87,1.04)  0.99 (0.91,1.08) |
